# Efficacy and safety of secukinumab in psoriasis: five-year real life experience^☆^

**DOI:** 10.1016/j.abd.2023.12.004

**Published:** 2024-07-19

**Authors:** Ersoy Acer, Hilal Kaya Erdoğan, Esra Ağaoğlu, Hatice Baştürk, Muzaffer Bilgin, Zeynep Nurhan Saraçoğlu

**Affiliations:** a Dermatology and Venereology Department, Eskisehir Osmangazi University, Eskisehir, Turkey; b Biostatistics Department, Eskisehir Osmangazi University, Eskisehir, Turkey

**Keywords:** Secukinumab, Efficacy, Safety, Smoking

## Abstract

**Background:**

The efficacy and safety of secukinumab in psoriasis patients has been demonstrated in randomized controlled clinical trials.

**Objectives:**

The authors aimed to evaluate the efficacy and safety of secukinumab in plaque psoriasis patients followed in our clinic.

**Methods:**

Data from 101 plaque psoriasis patients who received at least 16 weeks of secukinumab treatment between June 2018 and June 2023 were retrospectively analyzed.

**Results:**

Fifty-three (53%) of the patients were bionaive. PASI-75, -90, -100 response rates were 72%, 50%, 30% respectively at week 16 in all patients. PASI-75 and -90 responses were higher in naive patients at weeks 16 and 28 (p < 0.001, p < 0.001, p < 0.01, p = 0.01, respectively). The percentage of patients with PASI ≤ 1, ≤ 3, ≤ 5 were 50%, 77%, and 92%, respectively at week 16. They were higher in the naive group than in nonnaive group at weeks 16 and 28 (p = 0.02, p < 0.01, p = 0.05, p = 0.07, p < 0.01, p = 0.03, respectively). At week 52, PASI-75, -90, -100 responses were significantly lower in smoking patients (p = 0.04, p = 0.03, p < 0.01, respectively). The mean duration of secukinumab treatment was 19.80 ± 12.76 months. Secukinumab was discontinued 14 (26.4%) naive patients and 28 (58.3%) nonnaive patients at one occasion during treatment (p < 0.001). The most common adverse event in patients was mucocutaneous candida infection (8%). No hepatitis B or C reactivation and no active or reactivation tuberculosis were observed in any of the patients during the follow-up period.

**Study limitations:**

This is a single-center retrospective study with relatively few patients including only the Turkish population.

**Conclusion:**

Secukinumab seems to be effective in plaque psoriasis, particularly in bionaive and non-smokers. Moreover, it is safe in patients with inactive hepatitis or tuberculosis.

## Introduction

Psoriasis is a chronic, immune-mediated and common skin disease. It is estimated to affect approximately 125 million people worldwide.^1^ The pathogenesis of psoriasis is complex and not fully understood. However, there have been new developments in the pathogenesis of plaque psoriasis in recent years. A feed-forward inflammation mechanism, primarily the T-helper cell type 17 (TH17) pathway, has been shown to play a role in the pathogenesis. Thus, IL-17 antagonists have come into prominence in the treatment of psoriasis.^1^, ^2^ Secukinumab is a fully human monoclonal antibody against IL-17A and is the first agent against IL-17A. It was approved in 2015 for the treatment of moderate-to-severe psoriasis.^2^, ^3^

The efficacy and safety of secukinumab in psoriasis patients have been demonstrated in randomized controlled clinical trials.^4^, ^5^, ^6^ However, there may be significant differences between the results of clinical trials and those obtained from different populations in daily practice. In this study, the authors aimed to evaluate the efficacy and safety of secukinumab and factors that affected the efficacy of secukinumab in plaque psoriasis patients followed in our clinic.

## Materials and methods

In this observational, retrospective follow-up study, moderate-to-severe plaque psoriasis patients aged 18 years and older, who received at least 16 weeks of secukinumab in our clinic between June 2018 and June 2023 were included. Before secukinumab treatment, all infections including hepatitis B and C virus infection and tuberculosis (Tb) status are evaluated by history, physical examination, complete blood count, C reactive protein, transaminases, complete urinalysis, chest X-Ray studies, Purified Protein Derivative (PPD) test and/or QuantiFERON-TB tests and hepatitis B, C viruses markers, etc. Secukinumab (300 mg, s.c) was given as an initial loading dose at weeks 0, 1, 2, 3 and 4, and then once for every 4 weeks. Secukinumab injections were administered during the visits. The study was approved by the local ethics committee (decision nº 25/2023).

Sociodemographic characteristics, previous systemic and biological treatments, accompanying psoriatic arthritis and nail involvement, isoniazid and hepatitis B prophylaxis status of the patients were recorded. PASI scores were compared before secukinumab and at weeks 16, 28, 52, 76, 100, 148, and 196. The efficacy of secukinumab was expressed as the percentage of patients who achieved PASI-75, PASI-90 and PASI-100. In addition, patients who achieved absolute PASI ≤ 1, ≤ 3, ≤ 5 with secukinumab were determined as a percentage. Adverse effects, duration of secukinumab usage, and reasons for discontinuation secukinumab were recorded.

### Statistical analysis

The authors carried out a descriptive analysis of all of the variables included in the study. The quantitative variables were expressed as the mean ± Standard Deviation (SD), or as the median and range. The qualitative variables were expressed as an absolute value (n) and percentage.

Pearson Chi-Square and Independent Samples T-Test analyses were used in the analysis of the cross tables. IBM SPSS Statistics 21.0 (IBM Corp., Armonk, NY) was used for the analysis. For statistical significance, p < 0.05 was accepted as the criterion.

## Results

A total of 101 patients, 66 (65%) male and 35 (35%) female, were included in the study. The mean age of the patients was 50.07 ± 13.60 years. The mean Body Mass Index (BMI) of the patients was 28.7 ± 5.74. Seventy-four (73%) of the patients were overweight or obese (BMI ≥ 25). When the previous treatments were evaluated, 95 (95%) patients were treated with conventional systemic therapy and 53 (53%) patients were bionaive. Twenty-one (21%) patients had psoriatic arthritis, 38 (38%) patients had nail involvement (Table 1). Thirty-seven (37%) of the patients had at least one comorbidity. The most common comorbidities were hypertension (HT) (n = 19), diabetes mellitus (DM) (n = 13), thyroid diseases (n = 11), coronary artery disease (n = 4), asthma (n = 4), Parkinson's disease (n = 1), hemangioma (n = 1), migraine (n = 1), heart failure (n = 1), multiple sclerosis (n = 1), Buerger's disease (n = 1), rheumatoid arthritis (n = 1), Behçet's disease (n = 1), vitiligo (1), chronic renal failure (n = 1), polycythemia vera (n = 1).

**Table 1:** Baseline characteristics of study population.

**Number of patients, n (%)**	101 (100%)
**Gender, n (%)**	
Female	35 (35%)
Male	65 (65%)
**Age (years), mean ± SD**	50.07 ± 13.60 (20‒77)
**Smoking, n (%)**	56 (55%)
**Alcohol consumption, n (%)**	12, (12%)
**Mean duration of disease (year), mean ± SD**	19.85 ± 10.81 (1‒56)
**BMI, mean ± SD**	28.7 ± 5.74
**Over-weight or obese (BMI ≥ 25), n (%)**	74 (73%)
**Psoriatic arthritis, n (%)**	21 (%21)
**Nail psoriasis, n (%)**	38 (%38)
**PASI baseline, mean ± SD**	12.41 ± 6.47
**Previous systemic therapy, n (%)**	95 (95%)
Acitretin	43 (43%)
Methotrexate	89 (88%)
Ciclosporin	40 (40%)
NB-UVB	33 (33%)
**Previous biologic therapy, n (%)**	53 (52%)
Adalimumab	33 (33%)
Infliximab	13 (13%)
Etanercept	2 (2%)
Sertolizumab	3 (3%)
Ustekinumab	14 (14%)
**PPD test positivity, n (%)**	21 (37%) (Total 57 patient)
**QuantiFERON TB test positivity, n (%)**	19 (23%) (Total 83 patient)
**Isoniasid profilaxy**	48 (48%)
**Antiviral profilaxy for hepatitis B**	20 (20%)
**At least one comorbidity**	37 (37%)
**Mean duration of secukinumab usage (month)**	19.80 ± 12.76 (4‒52)
**Cause of discontinuing secukinumab n (%)**	
Primary ineffectiveness	6 (6%)
Secondary ineffectiveness	9 (9%)
Adverse effect and the others	4 (4%)
Unfollowed	21 (21%)

BMI, Body Mass Index; NB-UVB, Narrowband-Ultraviolet B, PPD, Purified Protein Derivative; SD, Standard Deviation.

**Figure 1:**
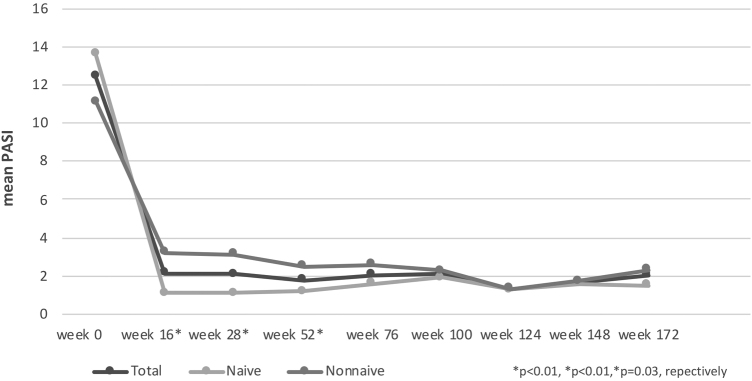
Average PASI improvement according to weeks. *Independent Samples T-Test.

**Figure 2:**
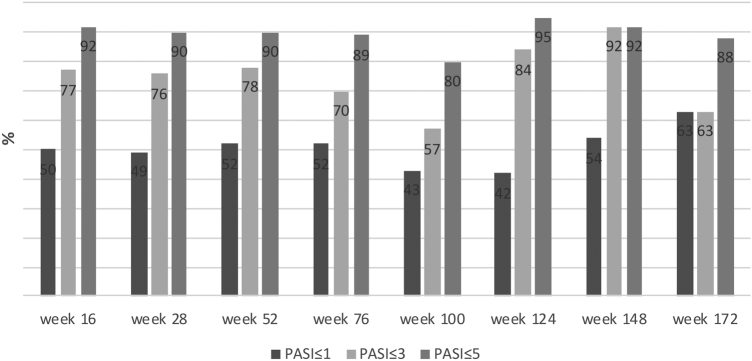
Percentage of achieved PASI ≤ 1, ≤ 3, ≤ 5 in all patients according to weeks.

The mean PASI of the patients was 12.41 ± 6.47 before secukinumab treatment. At 16, 28 and 52 weeks, the mean PASI was 2.11 ± 3.73, 2.08 ± 2.99, 1.79 ± 2.43, respectively. These were statistically significantly lower in bionaive patients compared to nonnaive patients at 16, 28 and 52 weeks (p < 0.01, p < 0.01, p = 0.03, respectively) (Fig. 1). At week 16, 72% of the patients had PASI-75, 50% had PASI-90, 30% had PASI-100. PASI-75 and -90 responses were higher in naive patients at weeks 16 and 28 (p < 0.001, p < 0.001, p < 0.01, p = 0.01, respectively) (Table 2). The percentage of the all patients with PASI ≤ 1, ≤ 3, ≤ 5 were in 50%, 77%, 92%, respectively at week 16 (Fig. 2). In naive group, these were 62.2%, 90.5%, 98.1% and 60%, 88.8%, 97.7% at weeks 16 and 28, respectively. In the nonnaive group, these were 37.5%, 62.5%, 85.4% and 38.1%, 61.9%, 80.9% at weeks 16 and 28, respectively. These were higher in naive group than nonnaive group (p = 0.02, p < 0.01, p = 0.05, p = .0.07, p < 0.01, p = 0.03, respectively).

**Table 2:** Results of PASI responses of patient groups according to weeks.

	Total	Naiv	Nonnaiv	p^a^
**Total patients, n (%)**	101 (100%)	53 (100%)	48 (100%)	
**Number of patients at week 16**	101	53	48	
PASI-75, n (%)	73 (72%)	47 (88.6)	26 (54.1%)	**p < 0.001**
PASI-90, n (%)	51 (50%)	35 (66.04%)	16 (33.3%)	**p < 0.001**
PASI-100, n (%)	30 (30%)	20 (37.7%)	10 (20.8%)	p = 0.10
**Number of patients at week 28**	87	45	42	
PASI-75, n (%)	63 (72%)	39 (86.6%)	24 (57.1%)	**p < 0.001**
PASI-90, n (%)	46 (53%)	30 (66.6%)	16 (38.1%)	**p = 0.01**
PASI-100, n (%)	22 (25%)	25 (33.3%)	7 (16.6%)	p = 0.12
**Number of patients at week 52**	67	36	31	
PASI-75, n (%)	47 (70%)	28 (77.7%)	19 (61.2%)	p = 0.23
PASI-90, n (%)	39 (58%)	24 (66.6%)	15 (48.3%)	p = 0.21
PASI-100, n (%)	24 (36%)	14 (38.8%)	10 (32.2%)	p = 0.76
**Number of patients at week 76**	46	25	21	
PASI-75, n (%)	32 (70%)	20 (80%)	12 (57.1%)	p = 0.17
PASI-90, n (%)	29 (63%)	17 (68%)	12 (57.1)	p = 0.65
PASI-100, n (%)	17 (37%)	9 (36%)	8 (38.1%)	p = 1.00
**Number of patients at week 100**	30	16	14	
PASI-75, n (%)	22 (73%)	12 (75%)	10 (71.4%)	p = 1.00
PASI-90, n (%)	16 (53%)	8 (50%)	8 (57.1%)	p = 0.98
PASI-100, n(%)	13 (43%)	6 (37.5%)	7 (50%)	p = 0.75
**Number of patients at week 124**	22	11	11	
PASI-75, n (%)	18 (82%)	10 (90%)	8 (72.7%)	p = 0.58
PASI-90, n (%)	12 (55%)	7 (63.4%)	5 (45.4%)	p = 0.67
PASI-100, n (%)	8 (36%)	4 (36.3%)	4 (36.3%)	p = 1.00
**Number of patients at week 148**	13	5	8	
PASI-75, n (%)	10 (77%)	5 (100%)	5 (62.5%)	p = 0.38
PASI-90, n (%)	6 (46%)	2 (40%)	4 (50%)	p = 1.00
PASI-100, n (%)	4 (31%)	2 (40%)	2 (25%)	p = 1.00
**Number of patients at week 172**	9	3	6	
PASI-75, n (%)	7 (78%)	3 (100%)	4 (66.6%)	p = 0.78
PASI-90, n (%)	6 (67%)	3 (66.6%)	4 (66.6%)	p = 1.00
PASI-100, n (%)	3 (33%)	1 (33.3%)	2 (33.3%)	p = 1.00
**Number of patients at week 196**	2	1	1	
PASI-75, n (%)	1 (50%)	1 (100%)	0 (0%)	p = 1.00
PASI-90, n (%)	1 (50%)	1 (100%)	0 (0%)	p = 1.00
PASI-100, n (%)	1 (50%)	1 (100%)	0 (0%)	p = 1.00

a Pearson Chi-Squared Tests.

In the present study, 56 (56%) of the patients were smoking. In general, response rates of secukinumab were low in patients with smoking at all weeks. At 52 weeks, PASI-75 (58.33% in smokers, 83.87% in non-smokers), PASI-90 (44.44% in smokers, 74.19% in non-smokers) and PASI-100 (19.44% in smokers, 54.84% in non-smokers) responses were statistically significantly lower in patients with smoking (p = 0.04, p = 0.03, p < 0.01, respectively). Moreover the percentage of patients with absolute PASI ≤ 1 at week 16 (39.29% in smokers, 64.44% in non-smokers) and with absolute PASI ≤ 3 at week 52 (66.67% in smokers, 90.32% in non-smokers) were statistically significantly lower in smokers (p = 0.02, p = 0.04, respectively). In this study, the mean duration of secukinumab treatment was 19.46 ± 12.49 months in smokers and 20.22 ± 13.23 months in non-smokers (p = 0.77). At one period of secukinumab treatment was discontinued for any reason in 48.21% of smokers and 33.33% of non-smokers (p = 0.19).

Twelve (12%) of the patients were drinking alcohol and there was no relationship between alcohol intake and treatment response rates (p > 0.05). In patients with BMI ≥ 25, the presence of arthritis, nail involvement, and at least one comorbidity, treatment response was generally low in all weeks compared to those without, but there was no statistically significant difference (p > 0.05).

The mean duration of secukinumab treatment was 19.80 ± 12.76 months. At the time point that the authors conducted the study, of all patients, 87 reached month 7, 67 reached month 13, 30 reached month 25, 22 reached month 31, 13 reached month 37, 9 reached month 43, and 2 reached month 49. Secukinumab was discontinued during one period of the treatment in 40 (40%) patients. Twenty-one (21%) of these patients did not want to use the secukinumab or dropped out of follow-up. Primary ineffectiveness in 6 (6%), secondary ineffectiveness in 9 (9%), adverse events in 2 (2%) patients. The adverse effects were neutropenia in one patient and diarrhea in one patient. Further, one patient developed breast cancer later and secukinumab was discontinued. One patient had worsening of psoriatic arthritis although his skin was good, secukinumab was discontinued. Secukinumab was discontinued in 14 (26.4%) of naive patients in 28 (58.3%) of nonnaive patients at one time of treatment (p < 0.001). The reasons for discontinuation of secukinumab in naive patients were unfollowed (n = 8, 53.8%), secondary ineffectiveness (n = 4, 30.7%) and adverse events (n = 2, 15.3%); in nonnaive patients, unfollowed (n = 14, 51.8%), primary ineffectiveness (n = 6, 22.2%), secondary ineffectiveness (n = 5, 18.5%), and other reasons (n = 2, 7.4%), respectively (p = 0.26).

The most common adverse event in patients was mucocutaneous candida infection (8%). Apart from this, mild upper respiratory tract infection in three patients, urinary tract infection in one patient, neutropenia in one patient, and diarrhea in one patient were observed.

In the present study, 20 (20%) of the patients had inactive Hepatitis B Virus (HBV) infection (hepatitis B core antibody (HbcAb)+, surface antigen of hepatitis B viruse (HBsAg)-, HBV DNA-). None of the patients had HBsAg+ and anti-hepatitis C virus (anti-HCV) antibodies+. All patients with inactive HBV infection received prophylaxis for reactivation by gastroenterology consultant. No hepatitis B or C reactivation was observed in the follow-up period. In the present study, PPD test was ≥ 5 mm in 21 (48%) of 57 patients evaluted with PPD test, and QuantiFERON-TB test was positive in 19 (23%) of 83 patients evaluated with QuantiFERON-TB tests. Fourty-eight (48%) of all patients received isoniazid prophylaxis by a pulmonary diseases consultant. No active tuberculosis or reactivation was observed in any of the patients during the follow-up period.

## Discussion

The efficacy and safety of secukinumab in psoriasis have been demonstrated in clinical trials. Response rates of PASI-75, -90, -100 at week 12 with 300 mg secukinumab were determined 81.6%, 59.2%, 28.6% /77.1%, 54.2%, 24.1 /86.7%, 55%, 26.7%, respectively in phase 3 randomized controlled trials.^4^, ^5^, ^6^ In this study, PASI-75, -90, -100 response rates were 72%, 50%, 30%, respectively at week 16 and they were lower than in clinical studies. In the present study, at 16 and 28 weeks PASI-75, -90, -100 response rates were higher in naive patients compared to nonnaive patients. In this study, 53% of the patients were bionaive. In the phase 3 randomized controlled trials, 71.4%, 88.4%, and 75% of patients were bionaive, respectively. Therefore, our PASI-75, -90, -100 response rates may have been lower than the clinical studies.^4^, ^5^, ^6^

In a real-life data set of 121 patients, PASI-75, -90, -100 response rates (84.3%, 68.6%, 18.2% vs. 89.3%, 73.6%, 9.1% vs. 86.1%, 64.6%, 7.6%) at week 16, 24 and 52 were higher than the present study. However, in this study, bionaive patients were quite (80%) higher than in this study.^7^ No previous biologic experience is one of the most important factors affecting the response rates to secukinumab in patients with psoriasis. As in clinical trials, it has been shown that no previous biologic experience is associated with a high response to secukinumab in many real-life datas.^7^, ^8^, ^9^, ^10^, ^11^, ^12^

In this study, absolute PASI of the patients was also evaluated with secukinumab treatment. The percentage of the patients with PASI ≤ 1, PASI ≤ 3, PASI ≤ 5 were 50%, 77%, and 92%, respectively at week 16. These rates were higher in naive patients than nonnaive patients. In another real-life data of 136 psoriasis patients treated with secukinumab, from Spain, the percentage of the patients with PASI ≤ 1, ≤ 2, ≤ 3, ≤ 5 were 47%, 64%, 75%, and 86%, respectively at week 16. These rates are lower than in this study. Similarly, higher response rates were reported in naive patients in this study. In this study, only 27.9% of patients were bionaive. This explains the lower response rates compared to the present study.^10^ In another study, low absolute PASI with secukinumab was associated with the presence of bionaivity.^11^

Extrinsic environmental factors such as alcohol intake, smoking, stress, sleep disturbance, sedentary life and diet affect the clinical presentation, severity, course and treatment response rates of psoriasis.^13^ In a study, it was found that obesity reduces the possibility of treatment with biologics.^14^ Moreover, higher BMI has been shown to be associated with a low response to secukinumab.^8^, ^10^ In another study, obesity was reported to be an important predictive factor for PASI-75 response rates with secukinumab at week 16.^7^ In the present study, PASI-75, -90, -100 response rates in all weeks were low in obese or overweight (BMI ≥ 25) patients, but there was no statistically significant difference.

Circulating proinflammatory mediators in psoriasis cause a widespread inflammatory response and comorbidities such as cardiovascular diseases, HT, DM, hyperlipidemia, metabolic syndrome (MS).^15^ Moreover, many diseases such as obesity, psoriatic arthritis, inflammatory bowel diseases, chronic kidney diseases, autoimmune thyroid diseases, mood disorders, and asthma may accompany psoriasis.^16^, ^17^ In this study, 37 (37%) of the patients had at least one comorbidity. The most common comorbidities were HT (19 patients), DM (13 patients), thyroid diseases (11 patients), coronary artery disease (4 patients), asthma (4 patients), respectively. Comorbidities also cause difficulties in the treatment of psoriasis. In a study, it was shown that comorbidities such as MS, HT, DM are associated with low response to secukinumab.^18^ In this study, secukinumab response rates were generally low in patients with comorbidities, but there was no statistically significant difference. Moreover, 52.35% of patients with comorbidities and 35.6% of patients without comorbidities discontinued secukinumab during one period of the treatment.

Fifty-six (56%) of the patients were smoking in this study. Response rates of secukinumab were generally low in patients with smoking at all weeks. The percentage of the patients with PASI ≤ 1 at week 16 and PASI ≤ 3 at week 52 was significantly lower in smokers. Moreover, PASI-75, -90, -100 response rates at week 52 were significantly lower in smokers. There are conflicting results associated with secukinumab response rates and smoking in the literature.^7^, ^18^ In a cohort study evaluating a total of 2384 patients receiving biological therapy including adalimumab, etanercept, infliximab, ustekinumab and secukinumab, it has been reported that smoking reduces the response rates to treatment with all agents.^14^ In this study, 12 (12%) of the patients were drinking alcohol and there was no relationship between alcohol intake and secukinumab response rates. This data was compatible with the literature.^7^, ^14^

A meta-analysis of 43 studies reported that adverse event rates were consistent with clinical studies and no new safety signal associated with secukinumab was seen.^19^ The most common adverse event was candida infections in real-life data with secukinumab. Adverse events such as nasopharyngitis, pneumonia, folliculitis, headache, diarrhea, subacute thyroiditis, neutropenia, thrombocytopenia, and elevated transaminases have been reported too.^7^, ^8^, ^9^, ^10^ The most common adverse event was oral candidiasis (8%) in the patients. Moreover, mild upper respiratory tract infection in three patients and urinary tract infection in one patient was observed. None of these patients required discontinuation of secukinumab. However, secukinumab was discontinued due to neutropenia in one patient and diarrhea in one patient. No inflammatory bowel disease (IBD) was detected in the control and follow-up period of the patient with diarrhea by the Gastroenterology consultant. In a real-life data of 108 psoriasis patients with treated secukinumab, diarrhea developed in three patients during the follow-up period, but none were found to have IBD.^20^ Further, a meta-analysis of 21 clinical studies including 7355 patients showed that secukinumab did not increase the risk of IBD.^21^ In the analysis of clinical trial and postmarketing surveillance data with up to five years, it was demonstrated that a low risk of malignancy for secukinumab.^22^ In the present study, breast cancer was detected in 1 patient during secukinumab treatment. The patient's family history was positive for breast cancer. Sekukinumab treatment was discontinued.

Twenty (20%) patients had inactive HBV infection in the present study. All received prophylaxis for reactivation by gastroenterology. No hepatitis B or C reactivation was observed in the follow-up period. Similarly, secukinumab has been reported to be safe in patients with a history of hepatitis B and C infection in real-life data.^9^, ^23^, ^24^ Secukinumab was reported to be safe in patients with tuberculosis in clinical trials and also in real-life data.^9^, ^23^, ^25^ In this study, 48 (48%) of all patients received isoniazid prophylaxis by the pulmonologist. No tuberculosis reactivation was observed in patients during the follow-up period.

In a meta-analysis of 43 studies, secukinumab survival was reported as 90% at 3 and 6 months, and 80% at 12 months.^19^ At the time point that the authors conducted the study, 87% of patients reached at 7 months, 67% at 13 months, 30% at 25 months, 22% at 31 months, 13% at 37 months, 9% at 43 month, 2% at 49 month. In 40 (40%) of the all patients, secukinumab was discontinued during one period treatment. Of these, 21 were unfollowed, 6 were discontinued due to primary ineffectiveness, 9 due to secondary to ineffectiveness, 2 due to adverse events (1 neutropenia, 1 diarrhea) 1 was due to breast cancer which appeared later and 1 due to worsening of arthritis (but skin was good). Part of the follow-up period of the patients coincided with the COVID-19 pandemic (2020‒2022), so the rate of patients who dropped out of follow-up may have been higher than in other studies. The proportion of patients who discontinued secukinumab due to ineffectiveness was similar to real-life data.^8^ However, in some studies, it was lower than in the present study. This may be due to the high rate of bionaive patients in these studies.^7^, ^11^ In one study, discontinuation of secukinumab due to ineffectiveness was higher than in this study. The rate of bionaive patients is very low in this study.^10^ Previous biologic experience is a very important factor for staying on secukinumab. In this study, discontinuation of secukinumab due to ineffectiveness was more common in nonnaive patients than in naive patients. Moreover, while secukinumab was not discontinued in any of the naive patients due to primary ineffectiveness, it was discontinued in 22.2% of the nonnaive patients.

### Limitations

The study has some limitations. This is a single-centre retrospective study and has relatively few patients and including only the Turkish population. But this study includes five-year real-life experiences of secukinumab in psoriasis. Despite these limitations, the authors believe that the results from the present study are valuable.

## Conclusion

Secukinumab is an effective treatment option in patients with plaque psoriasis in long-term use. No biological experience and no smoking may increase this effectiveness and survival in drugs. It is also a safe treatment option for hepatitis and latent tuberculosis reactivation.

## Financial support

None declared.

## Authors’ contributions

Ersoy Acer: Study concept and design; collection analysis and interpretation; statistical analysis; writing of the manuscript; critical review of the literature; approval of the final version of the manuscript.

Hilal Kaya Erdoğan: Study concept and design; collection analysis and interpretation; writing of the manuscript; critical review of the literature; approval of the final version of the manuscript.

Esra Ağaoğlu: Study concept and design; Collection analysis and interpretation; writing of the manuscript; critical review of the literature; approval of the final version of the manuscript.

Hatice Baştürk: Study concept and design; Collection analysis and interpretation; writing of the manuscript; critical review of the literature; approval of the final version of the manuscript.

Muzaffer Bilgin: Study concept and design; collection analysis and interpretation; writing of the manuscript; critical review of the literature; approval of the final version of the manuscript.

Zeynep Nurhan Saraçoğlu: Study concept and design; collection analysis and interpretation; writing of the manuscript; critical review of the literature; approval of the final version of the manuscript.

## Conflicts of interest

None declared.
